# Supplementary material for: Multi‐dataset identification of innovative feature genes and molecular mechanisms in keratoconus
Source: J Cell Mol Med. 2024 Sep 19;28(18):e70079. doi: 10.1111/jcmm.70079 (PMC11412914; doi:10.1111/jcmm.70079)
Supplement: Supplementary file 2 — Material S2. [file JCMM-28-e70079-s002.zip › 7.GSEA analysis/5.RNF217-AS1/KEGG_ENDOMETRIAL_CANCER.html]

Details for gene set KEGG\_ENDOMETRIAL\_CANCER[GSEA]

|  || Dataset | RNF217-AS1.group.cls#h\_versus\_l |
| Phenotype | group.cls#h\_versus\_l |
| Upregulated in class | l |
| GeneSet | KEGG\_ENDOMETRIAL\_CANCER |
| Enrichment Score (ES) | -0.4501674 |
| Normalized Enrichment Score (NES) | -1.7191525 |
| Nominal p-value | 0.01039501 |
| FDR q-value | 0.37124893 |
| FWER p-Value | 0.349 |
Table: GSEA Results Summary

  

Fig 1: Enrichment plot: KEGG\_ENDOMETRIAL\_CANCER      
 Profile of the Running ES Score & Positions of GeneSet Members on the Rank Ordered List

  

| SYMBOL | TITLE | RANK IN GENE LIST | RANK METRIC SCORE | RUNNING ES | CORE ENRICHMENT || 1 | PIK3R5 | na | 1058 | 0.434 | 0.0428 | No |
| 2 | BRAF | na | 5517 | 0.199 | -0.0522 | No |
| 3 | PIK3CD | na | 6778 | 0.159 | -0.0617 | No |
| 4 | ELK1 | na | 8380 | 0.119 | -0.0877 | No |
| 5 | SOS1 | na | 9467 | 0.096 | -0.1029 | No |
| 6 | GSK3B | na | 10962 | 0.071 | -0.1341 | No |
| 7 | PIK3CB | na | 11439 | 0.064 | -0.1370 | No |
| 8 | KRAS | na | 11445 | 0.064 | -0.1264 | No |
| 9 | CDH1 | na | 11891 | 0.057 | -0.1296 | No |
| 10 | PDPK1 | na | 12622 | 0.047 | -0.1428 | No |
| 11 | TCF7L2 | na | 12940 | 0.043 | -0.1447 | No |
| 12 | MAPK1 | na | 13362 | 0.038 | -0.1504 | No |
| 13 | EGF | na | 13646 | 0.034 | -0.1528 | No |
| 14 | PIK3CA | na | 14021 | 0.031 | -0.1584 | No |
| 15 | MAPK3 | na | 14127 | 0.029 | -0.1565 | No |
| 16 | CTNNA1 | na | 14215 | 0.028 | -0.1542 | No |
| 17 | ILK | na | 15326 | 0.016 | -0.1836 | No |
| 18 | AXIN1 | na | 15329 | 0.016 | -0.1810 | No |
| 19 | AKT2 | na | 15816 | 0.011 | -0.1932 | No |
| 20 | HRAS | na | 15878 | 0.010 | -0.1933 | No |
| 21 | PIK3R1 | na | 16145 | 0.007 | -0.1997 | No |
| 22 | MAP2K2 | na | 16236 | 0.006 | -0.2012 | No |
| 23 | BAD | na | 16311 | 0.006 | -0.2024 | No |
| 24 | TCF7L1 | na | 16424 | 0.004 | -0.2049 | No |
| 25 | NRAS | na | 17304 | -0.005 | -0.2294 | No |
| 26 | ERBB2 | na | 17478 | -0.006 | -0.2334 | No |
| 27 | AKT1 | na | 18280 | -0.013 | -0.2542 | No |
| 28 | CCND1 | na | 18820 | -0.019 | -0.2667 | No |
| 29 | RAF1 | na | 18969 | -0.020 | -0.2676 | No |
| 30 | FOXO3 | na | 19500 | -0.025 | -0.2786 | No |
| 31 | SOS2 | na | 19710 | -0.027 | -0.2801 | No |
| 32 | PTEN | na | 19758 | -0.028 | -0.2768 | No |
| 33 | CTNNB1 | na | 19818 | -0.028 | -0.2737 | No |
| 34 | TCF7 | na | 19939 | -0.029 | -0.2722 | No |
| 35 | CASP9 | na | 20912 | -0.039 | -0.2937 | No |
| 36 | GRB2 | na | 22374 | -0.055 | -0.3266 | No |
| 37 | PIK3R2 | na | 22894 | -0.061 | -0.3313 | No |
| 38 | MAP2K1 | na | 23215 | -0.064 | -0.3297 | No |
| 39 | EGFR | na | 24191 | -0.078 | -0.3447 | No |
| 40 | MLH1 | na | 24245 | -0.079 | -0.3329 | No |
| 41 | ARAF | na | 25299 | -0.095 | -0.3472 | No |
| 42 | CTNNA3 | na | 26243 | -0.111 | -0.3556 | No |
| 43 | CTNNA2 | na | 27584 | -0.139 | -0.3709 | No |
| 44 | PIK3CG | na | 30335 | -0.218 | -0.4135 | Yes |
| 45 | MYC | na | 30784 | -0.234 | -0.3870 | Yes |
| 46 | APC | na | 31337 | -0.256 | -0.3597 | Yes |
| 47 | AKT3 | na | 31832 | -0.281 | -0.3265 | Yes |
| 48 | TP53 | na | 32227 | -0.304 | -0.2865 | Yes |
| 49 | PIK3R3 | na | 32895 | -0.352 | -0.2463 | Yes |
| 50 | LEF1 | na | 34024 | -0.487 | -0.1967 | Yes |
| 51 | APC2 | na | 34163 | -0.517 | -0.1135 | Yes |
| 52 | AXIN2 | na | 34629 | -0.769 | 0.0029 | Yes |
Table: GSEA details [plain text format]

  

Fig 2: KEGG\_ENDOMETRIAL\_CANCER      
 Blue-Pink O' Gram in the Space of the Analyzed GeneSet

  

Fig 3: KEGG\_ENDOMETRIAL\_CANCER: Random ES distribution      
 Gene set null distribution of ES for **KEGG\_ENDOMETRIAL\_CANCER**
